# Molecular Characterization of *Anopheles sacharovi* Based on Sequences of ITS2-rDNA Region and COI Gene in North of Iran

**Published:** 2019-06-24

**Authors:** Sahereh Gholami, Hasan Bakhshi, Seyyed Hassan Moosa-Kazemi, Alireza Zahraei-Ramazani, Alireza Chavshin, Mohammad Mehdi Sedaghat

**Affiliations:** 1 Department of Medical Entomology, School of Public Health, Tehran University of Medical Sciences, Tehran, Iran; 2 Malaria and Vector Research Group, Biotechnology Research Center, Pasteur Institute of Iran, Tehran, Iran; 3 Department of Medical Entomology, School of Public Health, Urmia University of Medical Sciences, Urmia, Iran

**Keywords:** *Anopheles sacharovi*, *Anopheles martinius*, COI, ITS2-rDNA, Iran

## Abstract

**Background:**

Malaria is an important mosquito-borne disease considered as one of the public health concerns across many countries. *Anopheles* mosquitoes are the main vectors of *Plasmodium* parasites, which cause malaria. Some of these vectors such as *Anopheles maculipennis* s.l. and *Anopheles sacharovi* are considered as complex of sibling species distributed in north of Iran.

**Methods:**

This study was conducted in north and northwest of Iran including East Azerbaijan, West Azerbaijan, Ardabil, Golestan and North Khorasan provinces with emphasis on the northern borders of the country during 2015–2016. Adult specimens were collected and subjected to morphological identification as well as molecular analysis.

**Results:**

Overall, 10405 mosquitoes were collected comprising 21 species. *Culex pipiens* and *Cx. theileri* were found as the most frequent species in whole study area. Morphological identification showed that out of 1455 female *Anopheles* specimens, 77% belonged to *An. maculipennis* Group. Out of the identified species, ITS2 region and COI gene sequences of 8 *An. maculipennis* s.s. and 31 *An. sacharovi* representing all provinces were obtained and submitted to GenBank. The COI sequences for *An. sacharovi* revealed the presence of 9 haplotypes with similarity of 98.17–100%.

**Conclusion:**

Some investigations have reported *An. martinius* as a member of sibling species of *An. sacharovi* among Iranian *Anopheles* genus; while based on our study, there was no evidence of the presence of this species in north and northwest of Iran.

## Introduction

*Anopheles* mosquitoes are responsible for transmission of malaria parasites in humans. There are 30 definitive reported species, 3–4 biological forms and geographical races of *Anopheles* in Iran. There are seven primary malaria vectors recognized in Iran including *An. stephensi*, *An. culicifacies* s.l., *An. fluviatilis* s.l., *An. superpictus* s.l., *An. dthali*, *An. maculipennis* s.l. and *An. sacharovi* (1). Some of the most important species are in the Maculipennis group comprised as a primary or secondary vector of malaria parasites in the Palaearctic Region (2, 3).

There is a report on the bionomics of *An. maculipennis* and *An. sacharovi* from Iran and Iraq and the distribution of the two species in central and northern areas of Iran (4). *Anopheles maculipennis* s.l. was reported in central and northern areas of the country (5). Twenty-two species of *Anopheles* were listed in Iran based on literature records. The list included *An. martinius* among Iranian *Anopheles* species (6); although, there is no evidence of *An. martinius* occurrence in Iran so far.

As a result of recent molecular genetic studies, DNA sequence data are available for identification of the members of the complex. *Anopheles persiensis* was described as a new species of the *An. maculipennis* complex in north of Iran (2). Two members of *An. maculipennis* complex including *An. maculipennis* and *An. sacharovi* have been reported as vectors of malaria parasites in central and northern parts of the country. They are also considered as vectors of *Plasmodium* parasites from Armenia, Azerbaijan and Turkey (7). Members of the *An. maculipennis* complex are distributed mostly in northern and central areas of the country (8).

The *An. maculipennis* complex comprises several sibling species including major vectors of malaria parasites of historic Europe (9). Currently, there are 10 members of the *An. maculipennis* complex in the Palearctic Region including *An. melanon*, *An. messeae*, *An. persiensis*, *An. sacharovi*, *An. martinius*, *An. atroparvus*, *An. daciae*, *An. labranchiae* and *An. artemievi* (10). Out of the complex, six species including *An. maculipennis* s.s., *An. maculipennis*, *An. persiensis*, *An. messeae*, *An. atroparvus*, *An. labranchiae* and *An. sacharovi* have been identified in Iran based on molecular methods (3). *Anopheles melanoon* and *An. messeae* are listed as Iranian species based on the egg morphology studies as well as molecular studies (8).

This study was carried out based on the molecular and morphological characters of *An. maculipennis* s.l. collected from north and northwest of Iran with emphasis on border lines including East Azerbaijan, West Azerbaijan, Ardabil, Golestan and North Khorasan provinces where are considered as important biogeographic regions, being the corridor between Europe and Asia. These provinces have share borders with five countries including Turkmenistan, Armenia, Azerbaijan, Iraq and Turkey where the members of the *An. maculipennis* complex play an important role in malaria transmission. The potential occurrence of *An. martinius*, a close species of *An. sacharovi*, was also considered in this study.

## Materials and Methods

**Fig. 1.**
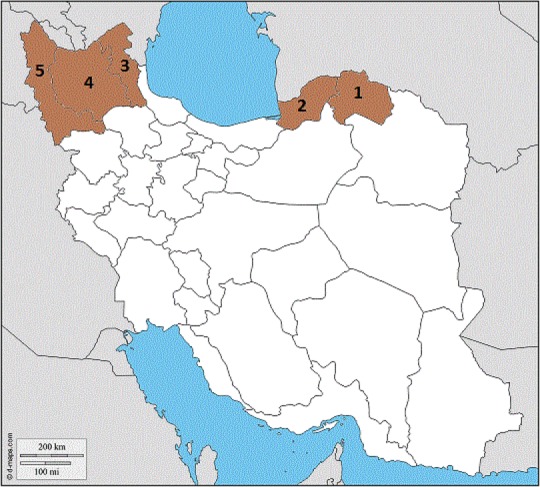
The study area: 1. North Khorasan, 2. Golestan, 3. Ardabil, 4. East Azerbaijan, 5. West Azerbaijan

Adult mosquitoes and larvae were collected from five provinces located in north of Iran during 2015–2016 using standard methods (Fig. 1, Table 1). Animal bite traps and shelter pit methods were used for collection of adults. The collection of larvae was carried out by dipping method.

**Fig. 2.**
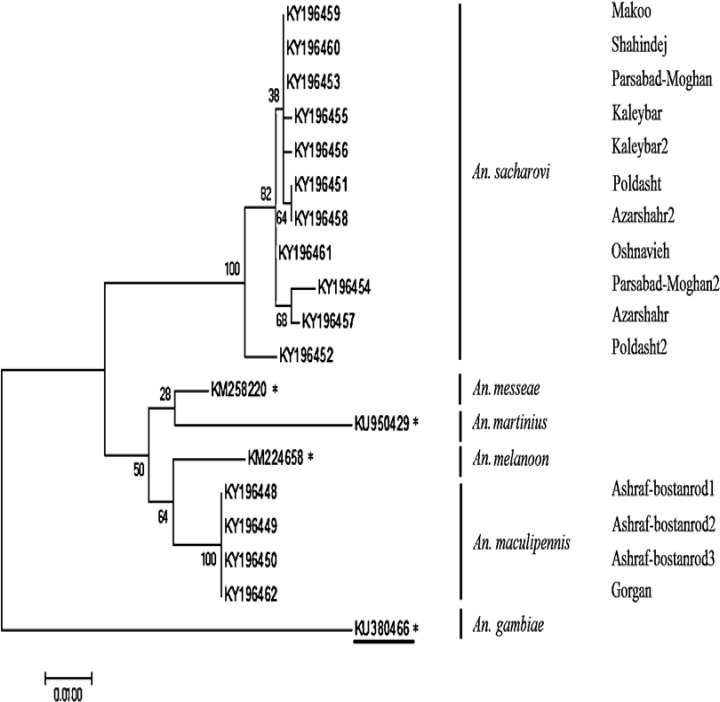
Phylogenetic tree constructed by 15 COI sequences (about 601bp) obtained from *Anopheles sacharovi*=11 and *Anopheles maculipennis*=4. The sequences KU950429: *Anopheles martinius*, KM224658: *Anopheles melanoon*, KM258220: *Anopheles messeae* and KU380466: *Anopheles gambiae* (as the out-group) were derived from GenBank

All samples were identified to the species level by using morphological keys (11). These identifications were used to target specimens for molecular identification using ribosomal internal transcribed spacer II (ITS2-rDNA) region and cytochrome oxydase I (COI) gene to differentiate cryptic species within the *An. maculipennis* complex. Genomic DNA of the mosquitoes was extracted using (G-spine ™) DNA Extraction Kit, according to the manufacturer’s instructions. Reactions were carried out in a total volume of 20µl using the PCR kit. The desired ITS2 fragments were amplified by using universal 5.8S (5´-TGTGAACTGC AGGACACATGAA-3´) as the forward and 28S (5´-ATGCTTAAATTAGGGGGTAGTC- 3´) as the reverse primers (12). The PCR conditions were as follows: 94 °C for 2min, followed by 25 cycles of 94 °C for 20sec, 50 °C for 15sec, and 70 °C for 25sec and terminating with a 72 °C for 5min. The desired COI fragments were amplified using LCO1490 (5´-GGTCAACAAATC ATAAAGATATTGG-3´) as the forward and HCO2198 (5´-TAAACTT CAGGGTGACC AAAAAATCA-3´) as the reverse primers (13). The PCR conditions were as follows: 94 °C for 2min, followed by 5 cycles of 94 °C for 30sec, 45 °C for 40sec, and 72 °C for 1min, followed by 35 cycles of 94 °C for 30sec, 55 °C for 30sec, and 72 °C for 1min, respectively, terminating with a 72 °C for 5min. Accuracy and quality of the amplicon were examined using a 1% agarose gel and visualized by Gel Doc after staining with Sinacolon® (Tehran, Iran) safe stain. DNA chromatograms were inspected using Chromas software (Version 2.23) and the sequences were submitted to GenBank. Similarity with other sequences in GenBank was assessed using BLAST (https://blast.ncbi.nlm.nih.gov/Blast.cgi) online tool. Phylogenetic tree was constructed by MEGA7 (ver. 7.0.21) software (Molecular Evolutionary Genetic Analysis) using Maximum Likelihood method with 1000 replicates of bootstrapping. Phylogenetic tree of fifteen COI sequences obtained from this study (*An. sacharovi*: 11 and *An. maculipennis*: 4) and four COI Genbank sequences as outgroup (KU950429: *An. martinius*, KM224658: *An. melanoon*, KM258220: *An. messeae* and KU 380466: *An. gambiae*) were created (Fig. 2).

## Results

### Morphological investigations

Out of 10405 specimens, 6556 adult samples and 3849 larvae were collected. Morphological identifications revealed that the specimens represented five genera and 21 species including *An. maculipennis* s.l., *An. sacharovi*, *An. claviger*, *An. hyrcanus*, *An. superpictus* s. l., *An. psudopictus*, *Culex hortensis*, *Cx. pipiens*, *Cx. theileri*, *Cx. modestus*, *Cx. mimeticus*, *Cx. perexiguus*, *Cx. tritaeniorhynchus*, *Aedes caspius*, *Ae. geniculatus*, *Ae. vexans*, *Ae. flavescens*, *Ae. echinus*, *Culiseta longiareolata*, *Cs. subochrea* and *Uranotaenia unguiculata*. *Culex pipien*s (30.2%), *Culiseta loniareolata* (23.8%) and *Cx. theileri* (22.3%) were the dominant species, and accounted for 76.3% of the collected samples (Table 2). *Culex pipien*s and *Cx. theileri* were found as frequent species in whole study area. *Uranotaenia unguiculata* (n=1, 0.009%), *Cs. subochrea* (n=4, 0.003 %) and *Ae. vexans* (n=4, 0.003%) were considered as *infrequent species*. *Uranotaenia unguiculata* was only collected in West Azerbaijan Province. Among *Aedes* mosquitoes, *Ae. caspius* (1.2%) was found in whole study area and *Ae. flavescens* in sympatry with *Ae. vexans* in Ardabil Province.

Among *Anopheles* mosquitoes, *An. claviger* and *An. hyrcanus* were widespread across the whole study area. *Anopheles maculipennis* s.l. (5.8%) was found in five provinces and *An. sacharovi* (0.3%) was found in sympatry with *An. maculipennis* s.l. only in three provinces located in northwest. *Anopheles superpictus* s.l. was also found with frequency of 3.9%. The last three species are the main malaria vectors in Iran.

### Molecular investigations

*Anopheles maculipennis* s.l. and *An. sacharovi* were subjected to molecular study using ITS2 and COI sequences. Eight sequences of *An. maculipennis* s.s. for the ITS2 region (n=4) and COI gene (n=4) were obtained and the sequences submitted to GenBank under accession numbers KY225560, KY225561, KY225562, KY225563 and KY196448, KY196449, KY 196450, KY196462 for ITS2 and COI regions respectively. All specimens were identified as *An. maculipennis* s.s. by an identity of 100 %. Thirty one sequences for *An. sacharovi* for the ITS2 region (n=19) and COI gene (n=12) were obtained and the sequences were submitted to GenBank under accession numbers KY225557-KY225559, KY263795-KY263806 and KY 196451-KY196461 for the ITS2 and COI regions respectively. All ITS2 sequences for *An. sacharovi* were identical with 100% similarity. The COI sequences for *An. sacharovi* showed that there were 9 haplotypes with similarity of 98.17–100%. The base composition of the COI fragments showed an AT bias with all sequences being between 66.4 and 67.8% AT rich (mean= 66.8%). Seventeen single nucleotide polymorphisms among the haplotypes were observed (Table 3).

Based on the COI sequences of *An. sacharovi* and *An. maculipennis* s.s. (about 601bp), phylogenetic tree was constructed. A tree for 15 COI sequences including 11 *An. sacharovi*, 4 *An. maculipennis* as well as 3 sequences of *An. maculipennis* complex deposited in GenBank (AN: KU950429 (*An. martinius*), KM 224658 (*An. melanoon*), KM258220 (*An. messeae*) and KU380466 (*An. gambiae*) was constructed (Fig. 2). The phylogenetic tree revealed the inter-population differences of the studied species. The smallest genetic distance was shown between the *An. sacharovi* populations from three provinces including East Azerbaijan, West Azerbaijan and Ardabil provinces rather than Golestan and North Khorasan provinces. The three mentioned provinces are closely located in northwest of the country (Fig. 1). Moreover, *An. melanoon*, *An. messeae* and *An. martinius* were recognized in separate clades from all *An. sacharovi* and *An. maculipennis* s.s. populations.

**Table 1.** Geographical coordinates of the study areas

**No.**	**Province**	**County**	**Latitude**	**Longitude**
**1**	West-Azerbaijan	Poldasht	39° 20′ 49.69″ N	45° 3′ 59.61″ E
		Shahindej	36° 40′ 26.74″ N	46° 34′ 12.48″ E
		Oshnavieh	37° 2′ 11.17″ N	45° 5′ 49.69″ E
		Makoo	39° 17′ 44.24″ N	44° 30′ 51.07″ E
**2**	Ardabil	Parsabad-Moghan	39° 37′ 14.80″ N	47° 54′ 18.22″ E
		Aslan-Duz	39° 26′ 29.57″ N	47° 24′ 40.25″ E
		Meshgin Shahr	38° 23′ 41.38″ N	47° 39′ 53.46″ E
**3**	East-Azerbaijan	Kaleybar	38° 51′ 51.27″ N	47° 2′ 25.94″ E
		Azarshahr	37° 44′ 39.54″ N	45° 59′ 13.95″ E
**4**	North-Khorasan	Bojnord	37° 28′ 12.74″ N	57° 18′ 51.61″ E
		Shirvan	37° 24′ 33.25″ N	57° 55′ 39.42″ E
**5**	Golestan	Gorgan	36° 50′ 44.31″ N	54° 26′ 21.61″ E

**Table 2.** Species composition of mosquitoes collected from East-Azarbaijan: EA, West-Azarbaijan: WA, Ardabil: A, Golestan: G, North-Khorasan: NKh

**No.**	**Species**	**Adults Species**					**Species Larvae**					**Adults (%)**	**Larvae (%)**	**Total (Adults and Larvae) (%)**

		**EA**	**WA**	**A**	**G**	**NKh**	**EA**	**WA**	**A**	**G**	**NKh**			
**1**	*An. maculipennis*	88	97	33	19	24	-	155	118	-	72	261 (3.98)	345 (8.96)	606 (5.8)
**2**	*An. sacharovi*	26	3	5	-	-	-	-	-	-	-	34 (0.51)		34 (32)
**3**	*An. claviger*	64	43	99	14	2	-	99	50	-	104	222 (3.38)	253 (6.57)	475 (4.56)
**4**	*An. hyrcanus*	12	-	23	20	9	-	-	-	-	-	64 (0.97)	-	64 (0.61)
**5**	*An. superpictus*	-	3	-	13	116	-	15	-	-	266	132 (2.01)	281 (7.3)	413 (3.96)
**6**	*An. psudopictus*	-	-	-	15	-	-	-	-	-		15 (0.22)	-	15 (0.14)
**7**	*Cx. hortensis*	120	9	-	20	3	-	180	106	-	28	152 (2.31)	314 (8.15)	466 (4.48)
**8**	*Cx. pipiens*	1326	9	63	109	46	-	336	1254	-	8	1553 (17.58)	1598 (41.51)	3151 (30.28)
**9**	*Cx. theileri*	1703	14	-	39	13	-	281	243	-	35	1769 (26.98)	559 (14.525)	2328 (22.38)
**10**	*Cx. modestus*	-	3	-	-	-	-	47	50	-	-	3 (0.04)	97 (2.52)	100 (0.96)
**11**	*Cx. mimeticus*	-	-	-	-	17	-	7	6	-	13	17 (0.25)	26 (0.67)	43 (0.41)
**12**	*Cx. perexiggus*	-	-	3	17	3	-	-	18	-	3	23 (0.35)	21 (0.54)	44 (0.42)
**13**	*Cx. tritarincus*	-	-	-	-	-	-	-	15	-	-	-	15 (0.38)	15 (0.14)
**14**	*Ae. caspius*	1	6	32	31	8	-	-	33	-	15	78 (1.18)	48 (1.24)	126 (1.21)
**15**	*Ae. geniculatus*	-	4	-	6	-	-	-	-	-	-	10 (0.15)	-	10 (0.096)
**16**	*Ae. vexans*	-	-	-	-	-	-	-	4	-	-		4 (0.1)	4 (0.038)
**17**	*Ae. flavecence*	-	-	-	-	-	-	-	11	-	-		11 (0.28)	11 (0.1)
**18**	*Ae. echinus*	-	-	-	15	-	-	-	-	-	-	15 (0.22)		15 (0.14)
**19**	*Cs. loniareolata*	2080	42	-	71	11	-	215	55	-	6	2204 (33.61)	276 (7.17)	2480 (23.83)
**20**	*Cs. subochrea*	-	-	-	4	-	-	-	-	-	-	4 (0.06)		4 (0.038)
**21**	*Ur. ungiuiculata*	-	-	-	-	-	-	1	-	-	-	-	1 (0.02)	1 (0.009)

**Total (%)**		5420 (52.09)	234 (2.24)	258 (2.48)	393 (3.78)	252 (2.42)		1336 (12.83)	1963 (18.86)	-	550 (5.3)	6556 (63)	3849 (37)	10405 (100)

**Table 3.** DNA sequence comparison of about 601bp of COI gene of *Anopheles sacharovi* distributed in the study area. Totally 9 haplotypes were identified within the sequenced samples, *: non-synonymous base change, Dots show identical sequences to the top sequence

**Position**	**38**	**116**	**155**	**170**	**203**	**209**	**278**	**323**	**326**	**341**	**347**	**368**	**401**	**487**	**509**	**557**	**563**
**Poldasht**	T	G	A	T	A	T	C	A	T	C	A	G	A	C	G	A	**A**
**Azarshahr**	.	.	.	.	.	.	.	.	.	.	.	.	.	.	.	.	**.**
**Parsabad-Moghan**	.	.	.	.	.	.	.	.	.	.	.	.	.	.	**T**	.	**.**
**Shahindej**	.	.	.	.	.	.	.	.	.	.	.	.	.	.	**T**	.	**.**
**Makoo**	.	.	.	.	.	.	.	.	.	.	.	.	.	.	**T**	.	**.**
**Kaleybar**	.	.	.	.	.	.	.	**G**	.	.	.	.	.	.	**T**	.	**.**
**Azarshahr**	.	.	**G**	.	.	.	.	.	.	.	**G**	.	.	.	.	.	**G**
**Poldasht**	**C**	**A**	.	**C**	**G**	**C**	**T**	.	.	.	**G**	**A**	.	.	**T**	**G**	**.**
**Kaleybar**	.	.	.	.	.	.	.	.	.	.	.	.	**G**	.	**T**	.	**.**
**Parsabad-Moghan**	.	.	**G**	.	.	.	.	.	**C**	.	**G**	**A**	.	G*	.	.	**.**
**Oshnavieh**	.	.	.	.	.	.	.	.	.	.	**G**	.	.	.	**T**	.	**.**
**Aslan-Duz**	.	.	.	.	.	.	.	.	.	**T**	.	.	.	.	**T**	.	**.**

## Discussion

The Maculipennis subgroup currently comprises 10 members including *An. artemievi*, *An. atroparvus*, *An. daciae*, *An. labranchiae*, *An. maculipennis*, *An. martinius*, *An. melanoon*, *An. messeae*, *An. persiensis* and *An. sacharovi*. Six members of *An. maculipennis* complex have been identified as primary or secondary vectors of malaria parasites in the Palearctic Region (2). These members are very close related species which are difficult to be identified by morphological characteristics. Although it is possible to distinguish *An. sacharovi* from other members by morphological characteristics, *An. martinius* is remained as a sibling species of *An. sacharovi* which makes it impossible to identify them by their morphological characteristics; these two species can be detected by cytological studies (14). Although the occurrence of *An. martinius* in Iran had been mentioned, there is no evidence for distribution of this species in the country so far. The most ambiguity is the distribution of *An. martinius* in east of Caspian Sea and east of Iran. Although it was reported in 1941 (15), but there is no new evidence of occurrence of this species in north of Tajikistan as well (16). This is in concordance with another study (17). Cytogenetic study showed that only *An. maculipennis* s.s. was present in this region. However, *An. artemievi* described as a homosequential species with *An. maculipennis*, could be erroneously identified as *An. martinius* (18). *Anopheles artemievi* is a new and predominate species in Kyrgyzstan, where it was identified as *An. martinius* (18). On the other hand, there are cytological or molecular evidences for occurrence of *An. martinius* in northeast of Turkmenistan, the Turkmen-Khorasan Mountain Range, Karakalpakstan and the Khorezm areas of Uzbekistan (19, 20).

Our molecular studies on the *An. maculipennis* complex were conducted to elucidate the possible occurrence of *An. martinius.* Members of the *An. maculipennis* complex were identified by sequence analysis of the ITS2-rDNA and COI gene. Morphological character-based identification showed that out of 1455 female *Anopheles* specimens, 1121 (77%) belonged to *An. maculipennis* complex. Molecular analysis of the complex indicated the presence of *An. sacharovi* and *An. maculipennis* s.s. in northwest and north of Iran. This result is in agreement with other studies (2, 3). Three genetically distinct species of the *An. maculipennis* complex were reported in Iran (2): *An. maculipennis* s.s., *An. sacharovi* and *An. persiensis*. However, the last species was not found in this study as it was found as a dominant species of the complex in the southern Caspian Sea littoral provinces of Guilan and Mazandaran (2). Six members of the group were reported based on molecular approach, while there was no *An. martinius* in the study area in northern Iran (21). No species of this complex was found but *An. maculipennis* s.s. based on molecular study in Zanjan Province located in the northwest of Iran (22). *Anopheles maculipennis* s.s. and *An. sacharovi* were found in nine provinces from northwest to central regions of Iran, it was no molecular evidence for presence of *An. martinius* either (23).

Molecular and phylogenetic analysis of the present investigation indicates more species diversity of *An. sacharovi* than has been recognized up to now. Divergence among the members of mosquito complexes varies but can be fundamental. Twenty-two species of *Anopheles* were reported in Iran including *An. martinius* (6). Apparently, there is no molecular evidence of *An. martinius* presence across the whole study area. The results correspond with other findings (24).

The base composition of the COI fragments showed an AT bias with all sequences being between 66.4% and 67.8% AT rich (mean=66.8%). These levels fall within the range of AT bias in mitochondrial genomes of other members of *An. maculipennis* complex including *An. maculipennis* (25), *An. messeae* (26), *An. sacharovi* (2, 3) and *An. martinius* (AN: KU950429). The COI sequences of *An. sacharovi* showed that there were 9 unique mtDNA haplotypes with similarity of 98.17–100%. The sequences were translated into amino acids to obtain the mitochondrial code. Translation of the sequences into amino acids showed all but one of the twelve specimens shared the same amino acid haplotype. Only one specimen (KY196454) from Parsabad-Moghan in Ardabil Province showed two nucleotide transversion (G⇔C, C⇔G) at the second and third codon position bases of 478 and 479bp (Table 3). These nonsynonymous bases change altered the codon from the consensus CCG (Arginine) to CGC (Proline), thus resulting in a unique amino acid haplotype.

## Conclusion

We collected three important malaria vectors in north of Iran. The permanent presence of historical vectors of pathogens results in potential epidemiological threats. Some malaria foci have been spotted in the northwest of Iran. Significant increases in commercial activities and travel from the neighbouring countries has led to increase concern about malaria and other vector-borne diseases in the Northern provinces. This has increased the concern of malaria cases occurrence in the area. A better understanding of the accurate identification of sympatric sibling species and their distributions remain important as the malaria control programs depend on the accurate identification of the vectors. Here, there is no evidence for occurrence of *An. martinius* in north of the country.
